# Optical Fiber Relative Humidity Sensor Based on a FBG with a Di-Ureasil Coating

**DOI:** 10.3390/s120708847

**Published:** 2012-06-27

**Authors:** Sandra F. H. Correia, Paulo Antunes, Edison Pecoraro, Patrícia P. Lima, Humberto Varum, Luis D. Carlos, Rute A. S. Ferreira, Paulo S. André

**Affiliations:** 1 Physics Department, University of Aveiro, 3810-193 Aveiro, Portugal; E-Mails: sandracorreia@ua.pt (S.F.H.C.); pantunes@ua.pt (P.A.); patriciapereira@ua.pt (P.P.L.); lcarlos@ua.pt (L.D.C.); 2 CICECO, University of Aveiro, 3810-193 Aveiro, Portugal; 3 Instituto de Telecomunicações, 3810-193 Aveiro, Portugal; 4 Instituto de Química, Universidade Estadual Paulista, Araraquara 14800-900, SP, Brazil; E-Mail: edison@iq.unesp.br; 5 Department of Civil Engineering, University of Aveiro, 3810-193 Aveiro, Portugal; E-Mail: hvarum@ua.pt

**Keywords:** fiber Bragg gratings, optical fiber sensors, relative humidity, organo-silica hybrid, structure health monitoring

## Abstract

In this work we proposed a relative humidity (RH) sensor based on a Bragg grating written in an optical fiber, associated with a coating of organo-silica hybrid material prepared by the sol-gel method. The organo-silica-based coating has a strong adhesion to the optical fiber and its expansion is reversibly affected by the change in the RH values (15.0–95.0%) of the surrounding environment, allowing an increased sensitivity (22.2 pm/%RH) and durability due to the presence of a siliceous-based inorganic component. The developed sensor was tested in a real structure health monitoring essay, in which the RH inside two concrete blocks with different porosity values was measured over 1 year. The results demonstrated the potential of the proposed optical sensor in the monitoring of civil engineering structures.

## 1. Introduction

The relative humidity (RH) of the air is defined as the ratio of the water vapor in the atmosphere to the saturation value. There are several methods to measure RH, including resistive, capacitive and hygrometric ones to be applied in distinct contexts [1]. Among the several solutions to sense RH, conventional electric sensors present several drawbacks such as high cost, need for maintenance and inability to be use in hazardous or explosive nature environments, in which electromagnetic interference immunity is required.

The optical fiber sensors can overcome these disadvantages, adding the possibility of multiplexing a large number of different sensors (temperature, displacement, pressure, pH value, humidity, high magnetic field and acceleration) into the same optical fiber, reducing the multiple cabling used in traditional electronic sensing [1]. In what concerns the RH sensors based on fiber-optic techniques, they can be classified according to the methods on which they rely. In particular, direct spectroscopy [2–9], evanescent wave [10–23], in-fiber grating [24–27] and interferometry [28–32].

The possibility of inscribing a Bragg grating in conventional optical fibers (FBG), opens a new field for optical sensing, being the subject of considerable research in recent years due to their advantages over conventional electronic devices. When used in structural monitoring, FBG sensors can monitor deformations and stresses in reinforced concrete elements, urban infrastructures, the curing process of concrete and mortars, RH, pH level, in geodynamic applications, among other variables and applications [33].

In this work we present an innovative RH sensor based on FBGs coated with an organo-silica hybrid material known as di-ureasil [34], which will be used to monitor the RH level in concrete blocks exposed to environmental conditions, demonstrating its ability to be used in structure health monitoring (SHM) applications.

This paper is organized as follows: after an introduction showing the requirements and advantages of optical fiber sensors, namely those based on FBG's, over conventional methods and devices, a brief description of FBG's and how they have been used as RH sensors is described in Section 2. The experimental procedure for the implementation of the proposed sensor is described at Section 3, with three sub-sections referring to the hybrid material production, the FBG coating and the sensor characterization. Section 4 shows the results of a real field application of this type of sensor in SHM. Finally, in Section 5, the main conclusions are presented.

## 2. Fiber Bragg Gratings: Fundamentals and RH Sensors

A Bragg grating is a passive optical device based on the refractive index modulation of the optical fiber core, created through the exposure of the fiber optics core to an optical interference pattern of ultraviolet radiation.

The operation principle of a FBG is based on the signal reflection in each plane of the periodic structure. When the Bragg condition is match, all the reflected components are in phase and are added, otherwise, the components are out of phase and vanished. The Bragg wavelength, *λ_B_*, at which the central wavelength of the reflection mode satisfies the first order Bragg condition, is given by:

(1) $$\lambda_{\text{B}}=2\;\Lambda \;{\text{n}}_{\text{eff}}$$

where *Λ* is the refractive index modulation period and *n_eff_* is the optical fiber core effective refractive index [33]. The Bragg wavelength depends both on the *n_eff_* and *Λ* and, therefore, any external disturbance acting on these parameters can be measured by analyzing the Bragg wavelength of the reflected signal. The application of a mechanical deformation or a temperature variation gives rise to a change in the refractive index and in the period values.

Several methods can be used to induce the fiber core refractive index modulation, being the most frequent ones the phase mask recording, the interferometric method, and the interferometric method with phase mask [33]. The latter one was selected to be used in the present work. A phase mask is an optical transmission element, containing a sequence of perturbations in the surface of a silica substrate, created by holographic processes or with lithographic scanning electron beam [35]. This inscription method is considered a major step forward in the recording of Bragg gratings in optical fiber due to its simplicity and ease of recording [36].

The usage of sensors based on FBGs to monitor RH requires the presence of a transducer layer that induces a thermal or mechanical actuation to which the FBG is directly sensitive [37]. In the proposed sensor the transducer layer is an organo-silica-based material with strong adhesion to the optical fiber that swells in the presence of distinct RH levels. The expansion of the material will be reflected in the FBG, resulting in a Bragg wavelength change. This deformation is also dependent on the Silica Young modulus and on the thickness of the material deposited on the fiber [38]. Furthermore, due to the thermal sensitivity of the FBG, it is required to include a thermal compensation scheme, to correct the environment effects. This can be easily solved by using an uncoated Bragg grating, thus insensitive to RH variations.

### 2.1. Related Works

The usage of a FBG in conjunction with a transducer layer to produce and optical sensor was already reported, using polymer-based materials as the swelling media. In particular, the influence of the RH on a FBG coated with a polymer was first described by Limberger *et al.* [39]. That sensor uses a polyimide coating as the transducer layer with a linear response over a wide range of values of RH (10–90%). Yeo *et al.* proposed a polyimide based RH sensor demonstrating that the sensor sensitivity is enhanced by increasing the polymer thickness from 10 to 42 μm [37]. However, in the latter case, the sensor response is slower and increases the sensitivity to temperature variations. A small degree of hysteresis was observed in all the tested situations. To overcome this problem, Venugopalan *et al.* inserted the FBG-polyimide optical sensor into a stainless steel tube containing multiple holes, enabling the contact with the surrounding environment [40]. To compensate the thermal effect on the RH sensor measurements, it was also included one uncoated FBG in the tube. This configuration provides mechanical resistance to the sensor, essential to withstand harsh conditions typically found in SHM and contributes to reduce the effect of cross-sensitivity to thermal changes [40]. Nigel *et al.* proposed a RH sensor based on a Pyralin coated optical FBG with a response time and resolution comparable to a capacitive RH sensor. A thin coating of ∼2 μm was used for a short response time [41]. A fast RH sensor was recently present by Mathew *et al.*, which can operate over a wide RH range, based on a bent single mode optical fiber [42]. The sensor makes use of an agarose hygroscopic coating with a linear response in the RH range between 25 and 90%. The response time of the sensor is about 50 ms, which is considerably faster. The same authors previously reported a miniature optical RH sensor based on a polymer infiltrated photonic crystal fiber interferometer [44,45]. A high sensitivity sensor to RH, with a variation in its reflected power of about 12 dB for a RH change of 84% was achieved [43]. The response time of the sensor is 400 ms for a RH modification of ∼30%.

The usage of FBG based sensors was also applied to SHM, in which the chemical attack of concrete structures may lead to a structural deterioration and moisture is one of the elements of the degradation mechanism. In 2006, Yeo *et al.* presented a fiber-optic-based RH sensor tested in the measurement of the moisture absorption in concrete [44]. The sensor uses an FBG coated with a moisture sensitive polymer. Several sensors were embedded in concrete samples with distinct different water/cement ratios and then immersed into water. It was found that the FBG sensors can be used to monitor the moisture content in different concrete samples, indicating new applications of this kind of sensors to ensure the integrity of civil engineering structures. In the same year, Yeo *et al.* presented a study of the detection of moisture ingress in concrete by monitoring the shift of the Bragg wavelength of two FBG-based RH sensors [45]. The used sensors presented sensitivities of ∼3.0× 10^‐3^ nm/%RH relying on a moisture-sensitive polymer layer coating.

The use of an organo-silica hybrid material provides further advantages to the optical sensor such as the improved interaction with the optical fiber and durability due to the presence of a silica-based inorganic skeleton that provides enhanced sensitivity and efficiency of the transduction process, besides the ease and low cost processing. Moreover, the di-ureasil organo-silica hybrid material is photosensitive to UV laser radiation without the need of photoinitioators [46], enabling the writing of a Bragg grating directly on the transducer layer.

In this context, the proposed optical sensor is presented as a low cost and low complexity tool for measuring RH that can be used in SHM, in particular for registering the RH level at points near the steel reinforcement of civil engineering structures, where high levels of RH can speed up the corrosion process. It can also be used in evaluating its overall health status, to identify the location and extent of any damage occurring. The proposed solution has application in monitoring of civil engineering structures (e.g., buildings, bridges, dams, monuments, among others), especially in large structures, where the ability to multiplex sensors becomes an advantage.

## 3. Experimental Section

### 3.1. Sensor Development

#### 3.1.1. Fiber Bragg Grating Production

The Bragg gratings were written in commercial photosensitive single mode fiber (FiberCore PS1250/1500) through the phase mask technique, using a KrF laser operating at 248 nm, with a pulse energy of 3 mJ, a repetition rate of 500 Hz and an exposition time of 10 s. The refractive index of the optical fiber core is 1.447 and we estimate a Gaussian refractive index apodization profile with a variation of *Δn*∼10^−4^ along the FBG length (∼3 mm). Four phase masks were used, producing FGBs with different Bragg wavelengths (*λ_B_* = 1537.9, 1539.8, 1543.3 and 1555.6 nm, Table 1), which allow the selective identification of all the sensors when multiplexed.

#### 3.1.2. Organo-Silica Based Materials Synthesis

The transducer layer is based on an organic-silica hybrid material, so-called di-ureasil, which is formed of polyether chains with average molecular weight of 600 g·mol^−1^ covalently linked to a siliceous inorganic skeleton by urea bridges [34], whose volume reversibly depends on distinct levels of RH. The di-ureasil, termed as d-U(600), was prepared using a procedure described in detailed elsewhere [46,47]. For the precursor synthesis, a tetrahydrofuran (THF, Riedel-de Haën) solution containing isocyanate-propyl-trietoxisilane (ICPTES, Aldrich) and a diamine commercially known as Jeffamine 600^®^ (Aldrich) is introduced into a reflux system, with a molar ratio Jeffamine 600^®^:ICPTES of 2:1 at 82 °C for 18 h, under magnetic stirring. The obtained suspension is transferred to a rotary evaporator to carry out the extraction of THF at 60 °C, under vacuum yielding to the non-hydrolyzed precursor (di-ureapropil-triethoxysilane, d-UPTES).

In order to promote the hydrolyze and condensation reactions of d-UPTES two distinct procedures were adopted to control the sol-gel reaction time. A fast sol-gel transition (∼40 s) was promoted adding ethanol (Fisher Chemical) to the d-UPTES in a 3:1 weight/volume ratio using a solution in ethanol of HCl 2.0 M (Aldrich) at a ratio of 13:1 (total volume of suspension/HCl). A slower sol-gel transition (∼5 h) takes place by changing the volume ratio suspension/HCl proportion to 20:1 and the concentration of HCl in order to delay the gelation, namely, 1.5 g of precursor d-UPTES is added to 0.5 mL of ethanol and 0.1 mL 1M HCl (in ethanol). The d-U(600) solutions with longer and slower gelification time will be deposit with controlled thickness using the dip-coating method.

#### 3.1.3. FBG and Organo-Silica Layers Assembling and Sensor Set Up

The average diameter of the deposited d-U(600) layers on the FBG was varied between 375 ± 5 μm and 591 ± 5 μm. The d-U(600) layers were deposited on the FBG using a homemade dip-coating system. The dip-coating process was performed at room temperature by immersing the FBG vertically in the d-U(600) solution at a velocity of 1.4 mm·s^−1^. This procedure was repeated 40 times with 120 s interval between depositions. During the deposition, the d-U(600) solution was kept under magnetic stirring to avoid premature gelation. After deposition, the fibers were transferred to the oven and kept at 50 °C for 40 h. In all the cases, the d-U(600) coating revealed strong adhesion to the optical fiber and mechanical stability. Hereafter, the sensors will be identified as *S_FBG-A_, S_FBG-B_, S_FBG-C_* and *S_FBG-D_*. Table 1 gathers selected optical and morphological features of the four developed sensors.

### 3.2. Sensors Characterization

**Figure 1.**
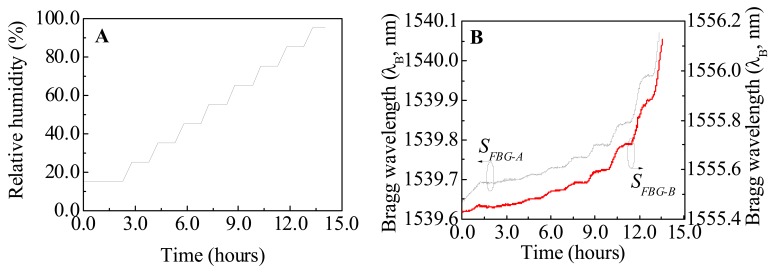
(**A**) Time evolution of the imposed relative humidity values within the thermal chamber characterization. The temperature was kept at a constant value of 30.0 °C. (**B**) Bragg wavelength response of the *S_FBG-A_* and *S_FBG-B_* RH sensors.

The implemented sensors were calibrated and tested under controlled temperature and RH values, using a thermal chamber (Model 340, *Challenge Angelantoni Industrie*) with a temperature and RH sensitivity of 0.1 °C and 0.1%, respectively. The thermal chamber undergoes a RH cycle with values between 15.0 and 95.0% (Figure 1(A)) at a constant temperature value of 30.0 °C. The sensors response to the imposed conditions is illustrated in Figure 1(B) for *S_FBG-A_* and *S_FBG-B_* RH sensors.

**Figure 2.**
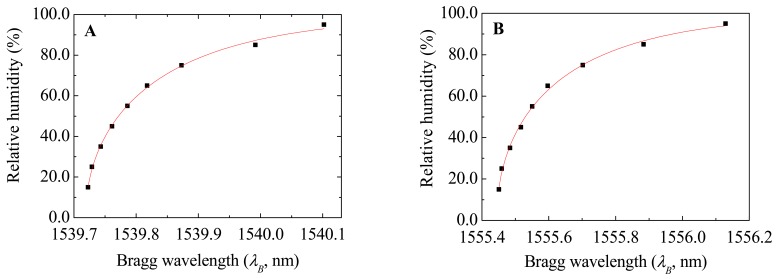
Bragg wavelength dependence on the relative humidity values at a constant temperature of 30.0 °C for the optical sensors (**A**) *S_FBG-A_* and (**B**) *S_FBG-B_*. The solid lines represent the data best fit using Equation (2) (R^2^ > 0.99).

For each HR step the Bragg wavelength was obtained in the steady state regime, revealing a growing nonlinear response as plotted in Figure 2, which is well described by a modified sigmoidal Richards function–type 1, given by:

(2) $$\text{RH}=\{\begin{array}{c} {\left[{\text{a}}^{1-\text{d}}-{\text{e}}^{-\text{k}(\lambda_{\text{B}}-(\lambda_{0}-S_{T}T))}\right]}^{1/(1-\text{d})},\text{d}<1\\ {\left[{\text{a}}^{1-\text{d}}+{\text{e}}^{-\text{k}(\lambda_{\text{B}}-(\lambda_{0}-S_{T}T))}\right]}^{1/(1-\text{d})},\text{d}>1 \end{array}$$

where *a, d, k* e *λ_0_* are fitting parameters, *S_T_* stands for the thermal sensitivity and *T* is the temperature at which the sensors are exposed. The parameter values obtained from the fit of the data in Figure 2 are listed in Table 2.

**Figure 3.**
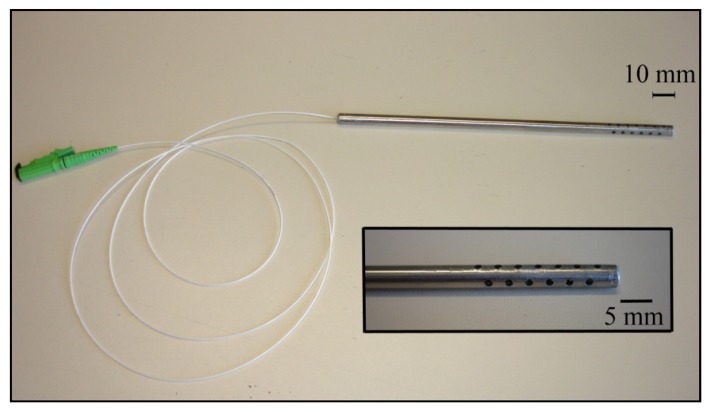
Prototype of the implemented RH optical sensor inserted in a protective stainless steel tube.

The sensors response is temperature dependent, due to the thermal expansion of fiber and coating, and to the thermo-optical properties of the optical fiber. Therefore, it is compulsory the compensation of the thermal effects on the sensors response. To protect the optical fiber, sensors *S_FBG-C_* and *S_FBG-D_* were encapsulated, being inserted into a stainless steel tube 15 cm long with an internal diameter of 2 mm, Figure 3. The tube has been punctured in the area of the coated optical fiber to allow the sensitive material to be exposed to the ambient humidity.

**Figure 4.**
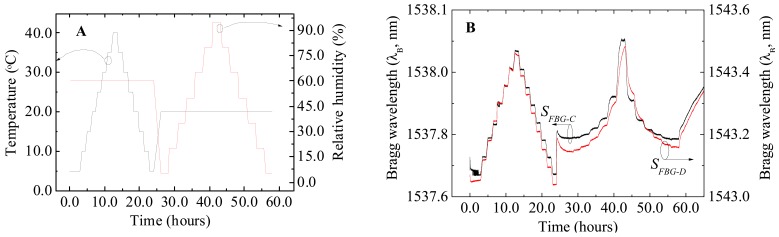
(**A**) Time evolution of the imposed conditions of temperature and relative humidity within the thermal chamber; (**B**) Bragg wavelength response for *S_FBG-C_* and *S_FBG-D_*.

For the temperature characterization, the thermal chamber undergoes a temperature cycle with values between 5.0 °C and 40.0 °C, at a constant RH of 60%. Afterwards another RH cycle with values between 5.0 and 95.0 % at a constant temperature value of 20.0 °C was imposed, Figure 4(A). The encapsulated sensors response to the imposed conditions is illustrated in Figure 4(B) for *S_FBG-C_* and *S_FBG‐D_* RH sensors.

**Figure 5.**
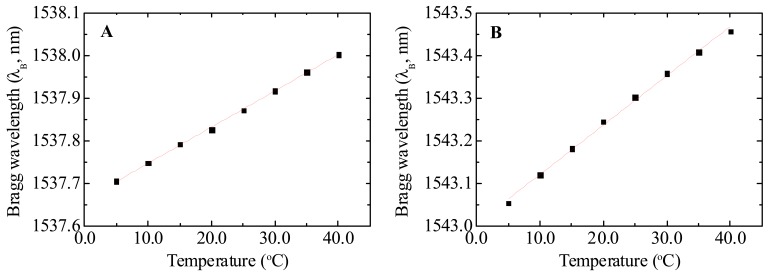
Bragg wavelength dependence on the temperature for (**A**) *S_FBG-C_* and (**B**) *S_FBG-D_* sensors. The solid lines represent the data best fit using a linear function (R^2^ > 0.99).

Figure 5 shows the variation of the Bragg wavelength on the temperature, revealing a linear dependence. From the data best fit, using a linear function, a thermal sensitivity (*S_T_*) of 8.48 ± 0.09 and 11.5 ± 0.25 pm·°C^−1^ was estimated for *S_FBG-C_* and *S_FBG-D_* sensors, respectively.

The Bragg wavelength dependence on the RH values for *S_FBG-C_* and *S_FBG-D_* are also well described by the modified Richards–type 1 [Equation (2)]. The dependence of the Bragg wavelength on RH is not linear in the entire RH range. Therefore, the sensor resolution will depend both on the RH level and on the resolution of the interrogation unit. Nevertheless, for higher relative humidity levels (>70%), we can assume a linear dependence of the Bragg wavelength with RH yielding to a sensitivity of 22.2 pm /%RH for sensor S_FBG-B_. This value is analogous to the highest reported value for optical sensors based on FBG [44].

Another important parameter of the sensor is the response time. Such response time was estimated, considering the worst case scenario, corresponding to a high HR value (85%), yielding to a value of 8.1 ± 1.1 and 16.4 ± 0.4 minutes for sensors S_FBG-A_ and S_FBG-B_, respectively. The attained values show that the sensors are suitable to assure the monitoring of RH, which has a time scale higher than the sensors response time. The reproducibility was tested with testing cycles, showing no hysteresis.

## 4. Sensor Application in Structure Health Monitoring

The applicability of the developed sensors was tested in a SHM essay. To accomplish this goal, relative humidity and temperature of two cubic (25 cm side) concrete blocks exposed to the environmental climatic conditions were monitored using the *S_FBG-C_* and *S_FBG-D_* sensors. The concrete blocks differ in the porosity being, expected, that the more porous one is more sensitive to changes of the relative humidity in the contour. In the preparation of the less porous concrete block (Block A) a 1:2.5:3 composition of Portland cement CEM II/L-C32,5N/river sand/gravel 8/12 was used. For the production of the more porous one (Block B) a 1:2.5:3 Portland cement CEM II/B-L32,5N/river sand/gravel 12/14 composition was used. It should also be noted that the second formulation of concrete was not vibrated during its manufacturing.

**Figure 6.**
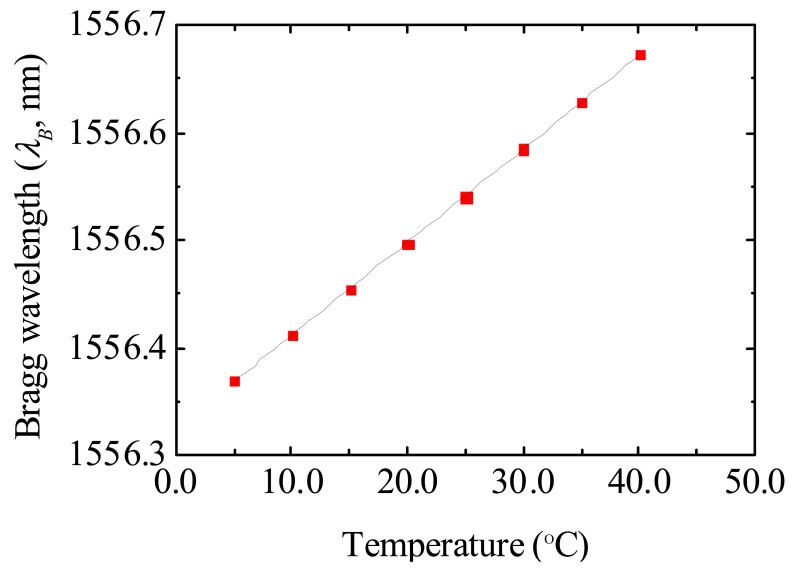
Bragg wavelength dependence on temperature variations for the FBG temperature sensor. The solid line corresponds to the data best fit using a linear function (R^2^ > 0.999).

Contrarily to the above mentioned testing and calibration conditions performed at a constant temperature, in the present situation it is expected that both temperature and relative humidity will change during the monitoring at ambient conditions. Therefore, knowing that the FBG-based sensors response is affected either by temperature and relative humidity changes, a temperature optical sensor was also constructed. This temperature sensor is also based on a FBG, thus allowing to be multiplexed with the developed *S_FBG-C_* and *S_FBG-D_* sensors. The uncoated FBG used to monitor temperature was placed inside a double needle, in order to protect the fiber from physical contact with the exterior, and simultaneously enable it to deform with the ambient temperature oscillations. The response of the temperature sensor to thermal variations is shown in Figure 6, revealing a linear dependence of the *λ_B_* value with temperature variations within 5.0 to 40.0 °C. The data was fitted using a linear function yielding to a temperature sensitivity of 8.67 ± 0.04 pm °C^−1^.

**Figure 7.**
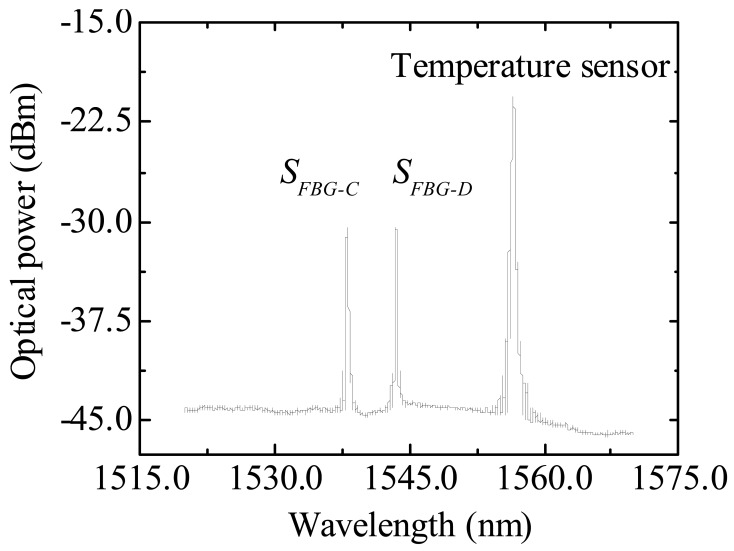
Optical spectra of the temperature and of *S_FBG-C_* and *S_FBG-D_* multiplexed sensors.

A single optical fiber was used to collect the signal from *S_FBG-C_* and *S_FBG-D_* sensors and the temperature sensor trough a 1 × 3 optical coupler that multiplexes the signal of the three sensors, Figure 7.

**Figure 8.**
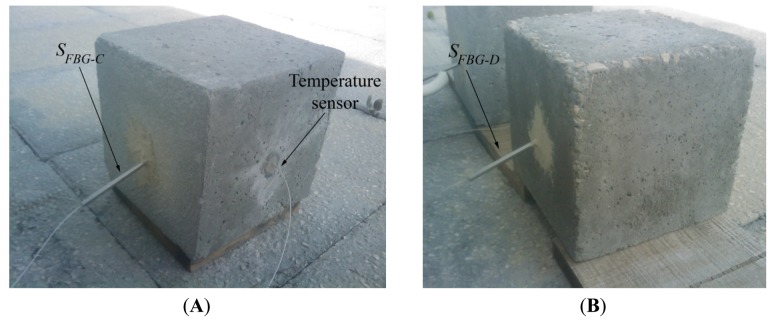
Photographs of the concrete blocks used in the structure health monitoring test: (**A**) Block A with *S_FBG-C_* and temperature sensor, and (**B**) Block B with *S_FBG-D_*. The blocks are cubic with 25 cm side.

The sensors were placed in holes made in the blocks so that the sensing portion (the Bragg grating) would be housed approximately at the center of the concrete block, after which the holes were sealed with epoxy resin (Figure 8). The Bragg grating wavelength was periodically measured for the selected *S_FBG-C_* and *S_FBG-D_* sensors and for the temperature one, using an optical commercial interrogation unit from Micron Optics, model sm125–500, with a wavelength resolution of 1 pm.

**Figure 9.**
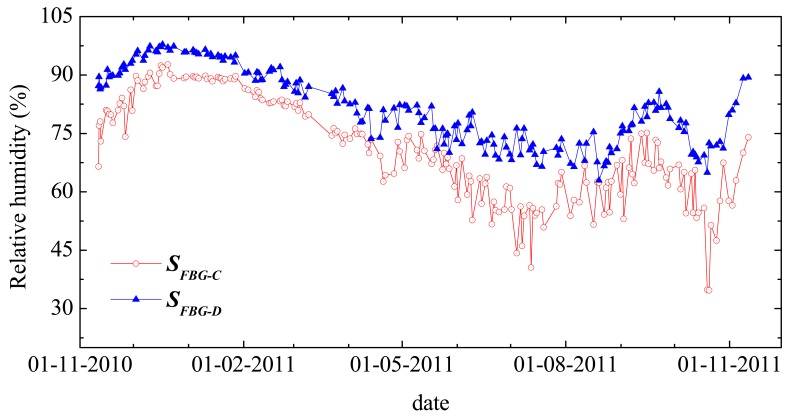
Relative humidity variation over time monitored with ***S****_FBG-C_* and ***S****_FBG-D_* sensors between 11 November 2010 and 11 November 2011.

Figure 9 shows the variation of RH over time using the developed sensors. As expected, the amount of moisture is more stable inside the concrete blocks than in the surrounding external environment.

The maximum RH value is measured during the months of December and January and the minimum one was monitored during August. The RH values measured with the *S_FBG-D_* sensor (Block B) are always higher than the ones measures with the sensor *S_FBG-C_* (Block A) due to the higher porosity of Block B, when compared with that of Block A. From the characterization data and from the test, it is concluded that the proposed di-ureasil based optical sensors provide a suitable response for the RH sensing in concrete structures with application SHM essays.

## 5. Conclusions

This work describes the development of a relative humidity optical sensor based on fiber Bragg gratings coated with an organo-silica di-ureasil hybrid material. The hybrid material was deposited by deep coating with controlled thickness and swells reversibly in the presence of distinct RH values (5.0–95.0 %) behaving as a transducer layer. When compared with polymer-based solutions, the proposed di-ureasil layer shows enhanced durability, sensitivity (22.2 pm/%RH), adhesion and compatibility with the optical fiber due to the presence of a siliceous based network. The produced optical sensors were tested in a real structure health monitoring essay, in monitoring the RH inside two concrete blocks for a time period of 1 year. The results demonstrated the viability of the produced sensors in the monitoring of civil engineering structures, namely, large structures where the ability to multiplex becomes an advantage.

## Figures and Tables

**Table 1.** Bragg wavelength (*λ_B_*) measured at 30 °C and RH value of 60% and d-U(600) layer average thickness (*W*) for the four developed sensors.

**Properties**	***RH sensors***			
	*S_FBG-A_*	*S_FBG-B_*	*S_FBG-C_*	*S_FBG-D_*
*W* (±5 μm)	485	591	375	450
*λ_B_* (nm)	1539.8	1555.6	1537.9	1543.3

**Table 2.** Fitting coefficients to Equation (2) for the *S_FBG-A_, S_FBG-B_, S_FBG-C_* and *S_FBG-D_* optical RH sensors.

**Coefficient**	***S_FBG-A_***	***S_FBG-B_***	***S_FBG-C_***	***S_FBG-D_***
*a* (%)	100	100	100	100
*λ_0_* (nm)	1541.5 ± 0.3	1558.6 ± 0.5	1539.1 ± 0.1	1544.1 ± 0.1
*d*	−1.1 ± 0.1	−1.1 ± 0.2	−1.6 ± 0.1	−0.7 ± 0.1
*k* (nm^−1^)	5.1 ± 0.4	3.1 ± 0.3	8.2 ± 0.5	7.1 ± 0.2

## References

[1] Yeo T.L., Sun T., Grattan K.T.V. (2008). Fibre-optic sensor technologies for humidity and moisture measurement. Sens. Actuators A Phys..

[2] Zhou Q., Shahriari M.R., Kritz D., Sigel G.H. (1988). Porous fiber-optic sensor for high-sensitivity humidity measurements. Anal. Chem..

[3] Posch H.E., Wolfbeis O.S. (1988). Optical sensors. 13. Fibre-optic humidity sensor based on fluorescence quenching. Sens. Actuators.

[4] Raichur A., Pedersen H. Fiber optic moisture sensor for baking and drying process control.

[5] Brook T.E., Taib M.N., Narayanaswamy R. (1997). Extending the range of a fibre-optic relative-humidity sensor. Sens. Actuators B Chem..

[6] Otsuki S., Adachi K., Taguchi T. (1998). A novel fiber-optic gas sensing arrangement based on an air gap design and an application to optical detection of humidity. Anal. Sci..

[7] Glenn S.J., Cullum B.M., Nair R.B., Nivens D.A., Murphy C.J., Angel S.M. (2001). Lifetime-based fiber-optic water sensor using a luminescent complex in a lithium-treated nafion (TM) membrane. Anal. Chim. Acta.

[8] Tao S.Q., Winstead C.B., Jindal R., Singh J.P. (2004). Optical-fiber sensor using tailored porous sol-gel fiber core. IEEE Sens. J..

[9] Bedoya M., Diez M.T., Moreno-Bondi M.C., Orellana G. (2006). Humidity sensing with a luminescent Ru(II) complex and phase-sensitive detection. Sens. Actuators B Chem..

[10] Russell A.P., Fletcher K.S. (1985). Optical sensor for the determination of moisture. Anal. Chim. Acta.

[11] Ogawa K., Tsuchiya S., Kawakami H., Tsutsui T. (1988). Humidity-sensing effects of optical fibers with microporous SiO_2_ cladding. Electron. Lett..

[12] Kharaz A., Jones B.E. (1995). A distributed fibre optic sensing system for humidity measurement. Sens. Actuators A Phys..

[13] Otsuki S., Adachi K., Taguchi T. (1998). A novel fiber-optic gas-sensing configuration using extremely curved optical fibers and an attempt for optical humidity detection. Sens. Actuators B Chem..

[14] Kharaz A.H., Jones B.E., Hale K.F., Roche L., Bromley K. (2000). Optical fibre relative humidity sensor using a spectrally absorptive material. Proc. SPIE.

[15] Bariain C., Matias I.R., Arregui F.J., Lopez-Amo M. (2000). Optical fiber humidity sensor based on a tapered fiber coated with agarose gel. Sens. Actuators B Chem..

[16] Gupta B.D., Ratnanjali (2001). A novel probe for a fiber optic humidity sensor. Sens. Actuators B Chem..

[17] Jindal R., Tao S.Q., Singh J.P., Gaikwad P.S. (2002). High dynamic range fiber optic relative humidity sensor. Opt. Eng..

[18] Muto S., Suzuki O., Amano T., Morisawa M. (2003). A plastic optical fibre sensor for real-time humidity monitoring. Meas. Sci. Technol..

[19] Arregui F.J., Ciaurriz Z., Oneca M., Matias I.R. (2003). An experimental study about hydrogels for the fabrication of optical fiber humidity sensors. Sens. Actuators B Chem..

[20] Gaston A., Perez F., Sevilla J. (2004). Optical fiber relative-humidity sensor with polyvinyl alcohol film. Appl. Opt..

[21] Alvarez-Herrero A., Guerrero H., Levy D. (2004). High-sensitivity sensor of low relative humidity based on overlay on side-polished fibers. IEEE Sens. J..

[22] Xu L.N., Fanguy J.C., Soni K., Tao S.Q. (2004). Optical fiber humidity sensor based on Evanescent-wave scattering. Opt. Lett..

[23] Corres J.M., Bravo J., Matias I.R., Arregui F.J. (2006). Nonadiabatic tapered single-mode fiber coated with humidity sensitive nanofilms. IEEE Photon. Technol. Lett..

[24] Kronenberg P., Rastogi P.K., Giaccari P., Limberger H.G. (2002). Relative humidity sensor with optical fiber Bragg gratings. Opt. Lett..

[25] Luo S.F., Liu Y.C., Sucheta A., Evans M., van Tassell R. (2002). Applications of LPG fiber optical sensors for relative humidity and chemical warfare agents monitoring. Proc. SPIE.

[26] Tan K.M., Tay C.M., Tjin S.C., Chan C.C., Rahardjo H. (2005). High relative humidity measurements using gelatin coated long-period grating sensors. Sens. Actuators B Chem..

[27] Konstantaki M., Pissadakis S., Pispas S., Madamopoulos N., Vainos N.A. (2006). Optical fiber long-period grating humidity sensor with poly(ethylene oxide)/cobalt chloride coating. Appl. Opt..

[28] Venugopalan T., Yeo T.L., Sun T., Grattan K.T.V. (2008). LPG based PVA coated sensor for relative humidity measurement. IEEE Sens. J..

[29] Mitschke F. (1989). Fiber-optic sensor for humidity. Opt. Lett..

[30] Arregui F.J., Liu Y.J., Matias I.R., Claus R.O. (1999). Optical fiber humidity sensor using a nano fabry-perot cavity formed by the ionic self-assembly method. Sens. Actuators B Chem..

[31] Kronenberg P., Culshaw B., Pierce G. (1999). Development of a novel fiber optic sensor for humidity monitoring. Proc. SPIE.

[32] Yu H.H., Yao L., Wang L.X., Hu W.B., Jiang D.S. (2001). Fiber optic humidity sensor based on self-assembled polyelectrolyte multilayers. J. Wuhan Univ. Technol..

[33] Antunes P., Lima H., Alberto N., Bilro L., Pinto P., Costa A., Rodrigues H., Pinto J.L., Nogueira R., Varum H., André P.S. (2011). Optical Sensors Based on Fiber Bragg Gratings for Structural Health Monitoring. New Developments in Sensing Technology for SHM.

[34] de Zea Bermudez V., Carlos L.D., Alcacer L. (1999). Sol-gel derived urea cross-linked organically modified silicates. 1. Room temperature mid-infrared spectra. Chem. Mater..

[35] Othonos A., Kalli K. (1999). Fiber Bragg Gratings: Fundamentals and Applications in Telecommunications and Sensing.

[36] Kashyap R. (2010). Fiber Bragg Gratings.

[37] Yeo T.L., Sun T., Grattan K.T.V., Parry D., Lade R., Powell B.D. (2005). Characterisation of a polymer-coated fibre bragg grating sensor for relative humidity sensing. Sens. Actuators B Chem..

[38] Antunes P., Lima H., Monteiro J., Andre P.S. (2008). Elastic constant measurement for standard and photosensitive single mode optical fiber. Microw. Opt. Technol. Lett..

[39] Limberger H., Giaccari P., Kronenberg P. Influence of Humidity and Temperature on Polyimide-Coated Fiber Bragg Gratings.

[40] Venugopalan T., Yeo T.L., Basedau F., Henke A.S., Sun T., Grattan K.T.V., Habel W. (2009). Evaluation and calibration of FBG-based relative humidity sensor designed for structural health monitoring. Proc. SPIE.

[41] David N.A., Wild P.M., Djilali N. (2012). Parametric study of a polymer-coated fibre-optic humidity sensor. Meas. Sci. Technol..

[42] Mathew J., Semenova Y., Farrell G. (2012). A fiber bend based humidity sensor with a wide linear range and fast measurement speed. Sens. Actuators A Phys..

[43] Mathew J., Semenova Y., Farrell G. (2012). Relative humidity sensor based on an agarose infiltrated photonic crystal fiber interferometer. IEEE J. Sel. Top. Quant..

[44] Yeo T.L., Eckstein D., McKinley B., Boswell L.F., Sun T., Grattan K.T.V. (2006). Demonstration of a fibre-optic sensing technique for the measurement of moisture absorption in concrete. Smart Mater. Struct..

[45] Yeo T.L., Cox M.A.C., Boswell L.F., Sun T., Grattan K.T.V. (2006). Monitoring ingress of moisture in structural concrete using a novel optical-based sensor approach. J. Phys. Conf. Ser..

[46] Vicente C.M.S., Venkatachaam R., Ferreira B.M., Marques P.G., Marques C.A.F., Pecoraro E., Carlos L.D., André P.S., Ferreira R.A.S. (2011). Thin film optimization design of organic-inorganic hybrids for waveguide high-rejection optical filters. Phys. Status Solidi..

[47] Fernandes V.R., Vicente C.M.S., Wada N., André P.S., Ferreira R.A.S. (2010). Multi-objective genetic algorithm applied to spectroscopic ellipsometry of organic-inorganic hybrid planar waveguides. Opt. Express.

